# Dental students’ preference and perception on intraoral scanning and impression making

**DOI:** 10.1186/s12909-021-02894-3

**Published:** 2021-09-22

**Authors:** Walter Yu-Hang Lam, Ken Chung-Kan Mak, Ebrahim Maghami, Pedro Molinero-Mourelle

**Affiliations:** 1 Prosthodontics, Restorative Dental Sciences, Faculty of Dentistry, The University of Hong Kong, 34 Hospital Road, Prince Philip Dental Hospital Sai Ying Pun, Hong Kong SAR, China; 2 Dental Service, Department of Health, 21/F, Wu Chung House, 213 Queen’s Road East, Hong Kong SAR Hong Kong, China; 3 Department of Mechanical Engineering and Mechanics, Drexel University, 3141 Chestnut Street, PA 19104 Philadelphia, USA; 4 Department of Reconstructive Dentistry and Gerodontology, School of Dental Medicine, University of Bern, 6 Hochschulstrasse, CHE 3012 Bern, Switzerland

**Keywords:** Education, Professional, Perception, Dental Impression Technique, Digital Technology, Education, Dental, Graduate, Graduate, Continuing

## Abstract

**Background:**

To investigate the preference and perception on intraoral scanning and impression making among dental students.

**Methods:**

Final-year dental students from the 2019 and 2020 cohorts were invited to complete an online questionnaire via Google-Form. Their preference on the intraoral-scanning/impression making techniques and their perception on these techniques including the ease of defect identification, ease of infection control, need of chairside support, ease to master the technique as a beginner, efficiency in their hands and ease to handle the scanner software (yes/no) were collected. The results were analysed using McNemar tests and binary logistic regression test. All tests were performed at significance level α = 0.05.

**Results:**

Ninety-seven students participated in this study with a response rate of 96.0 %. Eighty-one students (83.5 %) have tried intraoral scanning on peers. Fifty-three (54.6 %) students preferred intraoral-scanning and were categorized as *Pro-scanning group*. Forty-four (45.4 %) students either preferred impression-making (*n* = 21) or not sure (*n* = 23) were categorized as *Others*. More than half of students in both groups felt that intraoral-scanning is easier to identify defect, easier in infection control and require less chairside support. Higher proportion of students in the *Pro-scanning group* felt that intraoral-scanning requires less chairside support, easier to master as a beginner, more efficient in their hands and they can deal well with the scanner software than that in *Others* (*P* < 0.05). Regression shown that students preferred a technique that they perceived is more efficient (*P* = 0.000).

**Conclusions:**

While intraoral scanning has perceived advantages, many students still prefer impression making that works more efficient to them.

## Background

Digital technologies have been utilized to aid the disease diagnosis and management in dentistry. The use of intraoral scanning (IOS) for the Virtual Patient Models creation [1–4] and Computer-Aided Design/Manufacturing (CAD/CAM) of esthetic and high-strength all-ceramic restorations have been widely adopted in dental clinics [5, 6]. Improved patient comfort has been reported for IOS [7] and clinicians no longer need to pour the stone casts and wait for their setting [8]. Using the CAD/CAM workflow, the time required for dental restoration fabrications has been significantly reduced when compared to the conventional restorative work flow [9].

Despite the advantages of digital dentistry and CAD/CAM workflow, there are still significant proportion of dentists who do not adopt the IOS. One of the reasons is there are still some clinical limitations with these IOS systems, for instance, intra-arch discrepancy with IOS could be significant, rendering it not recommended for full arch prostheses in general [10]. Moreover, the sulcus reflection in the edentulous patient may be distorted during the scanning process and hence rendering it unsuitable for the fabrication of complete dentures with adequate border seal [11]. Moreover, there was huge variation in the types of procedures indicated for IOS among dental schools that have adopted IOS teaching [15].

Apart from clinical limitations, there are large variation in the reasons and barriers to adopt the IOS in the dental practice [12]. Factors such as innovation perception, personal, practice and social backgrounds all contribute to the acceptance to IOS. Graduating dental students therefore provides a relatively homogenous population for us to investigate the technology adoption of IOS. Compared to conventional impressions, training in dental schools in general devote less time on IOS, possibly due to time or equipment constraints [13, 14]. However, dental students’ acceptance in the intraoral scanning are expected to be highest, as they are more willing to learn new techniques in the dental school, and they should have minimum technical barrier in adopting digital equipment and software. Equipment cost is also not a consideration among dental students. It would be helpful to investigate student’s preferences and perceptions in IOS and impression making, which is less commonly explored.

In dental education, better learning experience will be achieved by considering students’ preference and perception when designing the dental curriculum [15–17]. However, students’ preference of IOS and impression making have not been investigated in relation to their learning experience [18]. Moreover, while the students’ preference or perception of IOS has been studied in dentistry [19–21], the combined survey of preference and perception allow more reliable assessments [22]. Therefore, the aim of the present study was to explore the preference and perception of students in IOS and impression making. The first null hypothesis was there is no difference in the perception of IOS and impression making among dental students who has different preferences. The second null hypothesis was there is no relationship between the preference and perception of IOS and impression making among dental students.

## Methods

This cross-sectional study was conducted in the academic year of 2018/2019 and 2019/2020 by means of an online questionnaire in the Faculty of Dentistry, The University of Hong Kong. This study were performed in accordance with the Declaration of Helsinki and has been approved by the Institutional Review Board of The University of Hong Kong and Hospital Authority Hong Kong West Cluster, Hong Kong (UW 20–514).

### Design/participants

The impression making was taught at the second-year of the sixth-year dental curriculum and was the mainstay clinical technique in patient management. As the dental teaching hospital, Prince Philip Dental Hospital, has been equipped with intraoral scanners, all final-year undergraduate dental students were required to attend to an intraoral scanning (IOS) course and were allowed to use IOS in patient management thereafter. The course content includes an introductory lecture by a faculty staff, a demonstration of scanning on a full dentate typodont model by product specialists from several scanner manufacturers (Trios 3, 3 Shape, Copenhagen, Denmark; iTero Element 5D, Align Technologies, California, United States; Medit i500, Medit Corp, Seoul, Korea; Primescan, Dentsply Sirona, Bensheim, Germany), followed by hands-on practice by students. If time permitted, there was a peer-practice on intraoral scanning.

After the course, students from two cohorts (Class of 2019 and 2020) were invited to complete an online questionnaire via Google Forms (Google Inc., California, United States). Written informed consents were obtained. The questionnaire includes the intraoral scanning or impression making preference and six questions regarding their perception on the clinical practicality of these techniques including the ease of defect identification, ease of infection control, need of chairside support, ease to master the technique as a beginner, efficiency in their hands and operating the scanner software (yes/no). The general meaning of “efficiency” was used in this study and there was no specific definition for this term.

### Statistical analyses

Students’ preference and perception of intraoral scanning and impression making were collected in proportions. Their differences were non-parametric nominal and paired in nature, and were analysed by the McNemer test. The relationship between preference (dependent variable) and perception (independent variables) of intraoral scanning and impression making was analysed by the binary logistic regression test. The level of significance was set at α = 0.05. Data were analysed using Statistical Product and Service Solution (SPSS) 27.0 (IBM Corp., New York, USA).

## Results

Ninety-seven final-year undergraduate dental students participated in this study, with 48 out of 50 students from Class of 2019 and 49 out of 51 students from Class of 2020. The response rate of this study was 96.0 %. The mean age of participated students were 23.1 and 60 students (61.9 %) were female and 37 students were male (38.1 %). Eighty-one students (83.5 %) had clinical experience in using intraoral scanners during the peer-practice.

### Preference of the intraoral scanning and impression making

Intraoral scanning was the preferred technique among more than half of participated students. Fifty-three students (54.6 %) preferred intraoral scanning, while 17 students (17.5 %) preferred conventional impression and 27 students (27.8 %) have no preference (Table 1). They were categorized into *Pro-scanning group* (n = 53) and *Others* (n = 44) respectively.

**Table 1:** Students’ preferred technique (*n* = 97)

	Number (%)
**Pro-scanning group**	
Prefer intraoral scanning	53 (54.6 %)
**Others**	
Prefer impression making	17 (17.5 %)
No preference	27 (27.8 %)

### Perception of the intraoral scanning and impression making

Students’ perception of intraoral scanning and impression making was presented in Table 2. More than half of participated students perceived that intraoral scanning were easier to identify a defect (n = 59, 60.8 %), easier in infection control (*n* = 68, 70.1 %), less chairside support (n = 83, 85.6 %), easier to master as a beginner (n = 66, 68.0 %), and more efficient in their hands (n = 51, 52.6 %). More than half of participated students perceived that they could operate the scanner software well (n = 55, 56.7 %).

**Table 2:** Perception of intraoral scanning and impression making by students

Perception	*Intraoral scanning*	*Impression making* ^a^	*Not sure* ^a^	*p-value* ^*^
**Easier to identify a defect**				
Overall	59	28	10	
*Pro-scanning group*	34	15	4	
				0.46
* Others*	25	13	6	
**Easier in infection control**				
Overall	68	19	10	
*Pro-scanning group*	39	10	4	
				0.41
* Others*	29	9	6	
**Less chairside support**				
Overall	83	9	5	
* Pro-scanning group*	45	8	0	
				0.03
* Others*	38	1	5	
**Easier to master as a beginner**				
Overall	66	21	10	
* Pro-scanning group*	41	10	2	
				0.03
* Others*	25	11	8	
**More efficient**				
Overall	51	31	15	
* Pro-scanning group*	41	9	3	
				0.000
* Others*	10	22	12	
Perception	*Yes*	*No* ^a^	*Not sure* ^a^	
**Dealt well with the scanner software**				
Overall	55	16	25	
* Pro-scanning group*	36	6	10	
				0.01
* Others*	19	10	15	

*McNemar tests

^a^Item “Impression making” and “No” were analysed with “Not sure” in the McNemar tests

Higher proportion of students in the *Pro-scanning group* perceived that intraoral scanning requires less chairside support (*P* = 0.03), easier to master as a beginner (*P* = 0.03), and more efficient in their hands (*P* = 0.000) than students in *Others*. Higher proportion of students in the *Pro-scanning group* perceived that they dealt well with the scanner software (*P* = 0.01) than students in *Others*.

### Relationship between the preference and perception of intraoral scanning and impression making

Binary logistic regression showed that students preferred a technique that they perceived to be more efficient in their hands (*P* = 0.000) (Table 3).

**Table 3:** Binary logistic regression of the preference (*Pro-scanning group* versus *Others*) and the perception of intraoral scanning and impression making

	B	S.E.	P-value	Exp (B)
Easier to identify a defect	-0.185	0.537	0.731	0.831
Easier in infection control	-0.236	0.588	0.688	0.791
Less chairside support	-2.155	1.218	0.077	0.116
Easier to master as a beginner	-0.235	0.617	0.703	0.791
More efficient	2.615	0.618	**0.000**	13.663
Dealt well with the scanner software	-0.652	0.522	0.212	0.521
Constant	2.452	2.822	0.385	11.610

## Discussion

More than half (54.6 %) of all participated final-year undergraduate students preferred intraoral scanning (IOS). This finding is similar to a previous study which found 63.9 % of students felt positive to IOS [23]. In the *Pro-scanning group*, there was a significantly higher proportion of students who perceived IOS requires less chairside support, easier to master as a beginner, and more efficient than the students in the *Others* (*P* < 0.05), the first null hypothesis was therefore rejected. Fifty-five (56.7 %) students in this study did not have difficulty in operating the scanner software which is in agreement with a previous study which found 60.2 % of students considered scanning process as manageable [23]. Considering these findings, the dental curriculum should incorporate more IOS component such as relevant background knowledge and clinical competence training.

In the binary logistic regression model, students’ preferred technique was linked to a technique that is perceived to be more efficient by them (*P* = 0.000). The second null hypothesis was therefore rejected. In the Oxford learner’s dictionaries, “efficient” means “doing something in a good, careful and complete way with no waste of time, money or energy” [24]. Lee et al. defined time and number of retakes/rescans as efficiency [25]. Among other clinical advantages, IOS is more efficient than impression making as it does not require time for mixing and setting of impression material, disinfecting the impression, and pouring of the stone models [26–28]. Areas that are less than ideal can be easily removed to allow recapture or simply re-scanning alone [29]. On the other hand, critical errors in the impressions (e.g. bubbles along preparation margins, unclear margins) will render the whole impression useless and the whole impression will need to be made again [30]. Such repeated attempts of impressions are common [31, 32], especially among dental students who are still in the learning stage. With the aid of intraoral scanners, errors in impression can be identified easily under the magnified view and any further corrections can be made much more efficiently, saving student’s chairside time [19, 23, 33, 34].

In the present study around one-fifth (17.5 %) of students still settled upon impression making and around one-fourth of students (27.8 %) did not have any preferred technique. We also found that the particular technique perception that works more efficient might affect the preference of students, therefore, with more practise and clinical exposure of IOS, more students might prefer IOS. This has implication that more IOS courses, integrated in the postgraduate degrees or as short continuous professional development modules, should be provided to the dental professionals. On the other hands, as was mentioned in the introduction, the full arch scanning error and the sulcus capture failure mean that the teaching of impression making and related knowledge should not be abandoned at this level. With these perceived advantages, many undergraduate students still like to choose impression making and realized that impression making is more efficient in their hands. Nevertheless further studies are needed in order to determine the factors that affect students’ perceived technique that is more efficient to them.

Intraoral scanners, as with any impression technique, processes a learning curve [35, 36]. Some areas in the mouth might not be easy to capture with intraoral scanners such as the distal surfaces of the last tooth in an arch as well as the proximal surfaces near to a bounded saddle. It is difficult for beginners to move the tip of intraoral scanners around while following the scanner’s signals based on recognizing previously captured surfaces [37]. The IOS and impression making probably require different competence skills [25, 34, 38]. Students in this study were exposed to various intraoral scanners, differences in the system and the scanning protocols may result in variations in success of impression making [37, 39, 40]. However, most of the students able to operate the scanners well during the hands-on and peer-practice. The user-friendliness of the newer generation of intraoral scanners might be the key factor for students’ preference and their final assessment as well.

Some limitations of this study include the lack of clinical experience of students in intraoral scanning for a fair comparison. The lack of a real patient practice should be considered since a clinical scenario could provide additional information considering the limitations and complications of impression making in dental students at the beginning of their clinical practice. Students’ only experience with intraoral scanner might be the peer-practice that is straightforward because their colleagues usually have a full set of dentition. Participated students in this study were young and were expected to more adaptive to new technology such as IOS. The result of this study may be extrapolated to dentists who have more clinical experience and should be more efficient in impression making, therefore higher proportion of them is expected to prefer impression making than IOS. Further studies are needed to investigate the preference and perception of dentists in IOS and impression making.

## Conclusions

Intraoral scanning (IOS) is favoured among final-year undergraduate dental students. While intraoral scanning has perceived advantages, many students still prefer impression making that works more efficient to them. More IOS courses, integrated in the postgraduate degrees or as short continuous professional development modules, should be provided to the dental professionals. Meanwhile, dental schools should prepare students to be competent in both techniques to handle different clinical situations.

## Data Availability

The dataset used and/or analysed is this study are available from the corresponding author on reasonable request.

## Abbreviations

IOS: Intraoral Scanning
CAD/CAM: Computer-Aided Design/Manufacturing
